# Autoimmune Lymphoproliferative Syndrome and Epstein-Barr Virus-Associated Lymphoma: An Adjunctive Diagnostic Role for Monitoring EBV Viremia?

**DOI:** 10.1155/2013/245893

**Published:** 2013-07-25

**Authors:** Romina Pace, Donald C. Vinh

**Affiliations:** ^1^Department of Medicine, McGill University Health Centre, Montreal, QC, Canada H3G 1A4; ^2^Division of Infectious Diseases, Division of Allergy & Clinical Immunology (Department of Medicine), Department of Medical Microbiology, Department of Human Genetics, McGill University Health Centre, Montreal General Hospital, 1650 Cedar Avenue, Rm A5-156, Montreal, QC, Canada H3G 1A4

## Abstract

*Background*. Autoimmune lymphoproliferative syndrome (ALPS) is a genetic disorder of lymphocyte homeostasis due to defects in FAS-mediated apoptosis. ALPS is characterized by childhood onset of chronic lymphadenopathy and splenomegaly, autoimmunity, an expanded population of double-negative T cells (DNTCs), and an increased risk of lymphoma. This propensity for lymphoma in ALPS is not well understood. It is possible that lymphomagenesis in some of these patients may result from Epstein-Barr virus (EBV) infection exploiting the defective T-cell surveillance resulting from impaired FAS-mediated apoptosis. *Case Presentation*. We report the first case, to our knowledge, of lymphoma in a patient with ALPS that was clinically heralded by progressively increasing EBV viremia. We discuss its practical implications and the possible immune pathways involved in the increased risk for EBV-associated lymphoproliferative disorders in ALPS patients. *Conclusion*. In patients with ALPS, distinguishing chronic lymphadenopathy from emerging lymphoma is difficult, with few practical recommendations available. This case illustrates that, at least for some patients, monitoring for progressively increasing EBV viremia may be useful.

## 1. Background

Autoimmune lymphoproliferative syndrome (ALPS) is a Mendelian disorder of lymphocyte homeostasis caused by defects in FAS-mediated apoptosis [1]. The majority of cases to date are due to heterozygous germline mutations in the *APT1* (*TNFRSF6*) gene, which encodes the cell surface-expressed transmembrane receptor, FAS (CD95) [2]. Ligation of FAS by its cognate ligand (FAS ligand; FAS-L) triggers B- and T-lymphocyte apoptosis. Mutations in FAS, FAS-L, or in the downstream molecules that constitute the apoptosis-inducing complex (i.e., Caspase-10) impair programmed cell death of lymphocytes. Consequently, ALPS is characterized by childhood onset of chronic (>6 months) noninfectious, nonmalignant lymphadenopathy and splenomegaly, associated with an increased circulating number of a unique T-cell population that is CD3+ and expresses the *α*/*β* receptor but is CD4− and CD8−, the so-called double-negative T cells (DNTCs). DNTCs are considered elevated when they are >1.5% of total lymphocytes or >2.5% of CD3+ lymphocytes in the setting of normal or elevated lymphocyte counts [2]. Additional diagnostic criteria for ALPS include polyclonal hypergammaglobulinemia, elevated vitamin B12 levels, and elevated immune biomarkers in plasma (e.g., soluble FAS-L, IL 10, and IL-18) [2]. The clinical course of ALPS is marked by increased rates of autoimmunity and of malignancy. In particular, individuals with germline FAS mutations have risks for Hodgkin's lymphoma (HL) and non-HL of 51- and 14-fold, respectively, above the general population [3]. This predisposition to lymphoma in ALPS is not fully understood. Failure of the FAS apoptotic pathway is apparently necessary, likely by providing an expanded lymphoid pool at risk for clonal transformation. However, the temporal delay between the onset of ALPS manifestations and that of lymphoma suggests a requirement for additional oncogenic events. Epstein-Barr virus (EBV) is a ubiquitous lymphotropic *γ*-herpesvirus. With its ability to establish lifelong latency in B cells and its growth transformation capacity, EBV is associated with various lymphoproliferative disorders (LPD). Diagnoses of some EBV-related LPD are facilitated by quantitative monitoring of EBV viral load (VL) in blood. We report the first case, to our knowledge, of lymphoma in a patient with ALPS that was heralded by progressively increasing EBV viremia and discuss its clinical implications.

## 2. Case Presentation

**Figure 1:**
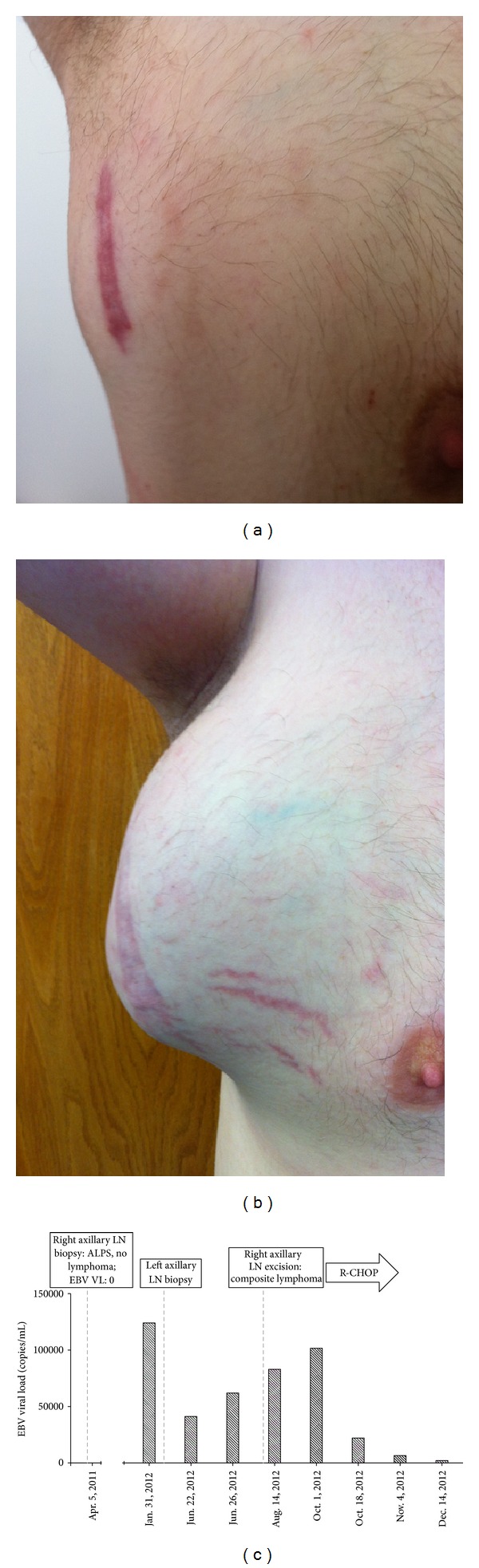
EBV viremia and EBV^+^ lymphoma in ALPS. A 33-year-old male with ALPS-Ia developed asymptomatic worsening lymphadenopathy (Right axillary lymph node in July 2011 (a) and June 2012 (b)), with progressively increasing EBV viral load (VL). He was found to have a composite EBV^+^ lymphoma. (c) Monitoring of whole blood EBV VL demonstrates an increasing viremia with concomitant identification of lymphoma and improvement in viremia with (rituximab, cyclophosphamide, doxorubicin, vincristine, and prednisone; R-CHOP) chemotherapy.

A 33-year-old French-Canadian male was referred for reevaluation of chronic lymphadenopathy and splenomegaly since the age of 4, when he had been diagnosed with “chronic EBV illness” based on serology. He had no history of unexplained fever, liver dysfunction, cytopenia, mosquito bite reactions, or hydroa vacciniforme, features that would suggest the nosologic entity “chronic active EBV.” Based on elevated serum vitamin B12 level, polyclonal hypergammaglobulinemia and 15% DNTC, the diagnosis of ALPS was confirmed by identification of a heterozygous c.784A>T mutation in *TNFRSF6*, resulting in p.I262F mutation in the intracellular death domain of FAS (GeneDx). Initial whole blood EBV VL was 0 copies/mL. Baseline positron-emission tomography (PET-CT) demonstrated diffuse hypermetabolic lymphadenopathy with enlarged but not hypermetabolic spleen. Right axillary lymph node biopsy revealed paracortical expansion with DNTCs and increased number of EBV^+^ B cells (by EBV-encoded small RNA (EBER)) but no lymphoma. At 1-year followup, he was noted to have asymptomatic EBV viremia (Figure 1). Serial monitoring of the viral load demonstrated an initial decline and then progressive increase in EBV viremia followed by worsening lymphadenopathy, without constitutional symptoms. PET-CT demonstrated nodal foci with significantly increased metabolic activity. Lymph node excision *in toto* was required because of a nondiagnostic percutaneous biopsy; histopathological analysis and molecular rearrangement studies demonstrated a composite lymphoma (i.e., follicular lymphoma, diffuse large B-cell lymphoma (DLBCL), and classical HL). Chemotherapy with Rituximab, to target the EBV^+^ B cells, cyclophosphamide, doxorubicin, vincristine and, prednisone (R-CHOP) has led, to a dramatic improvement in his lymphomas and concomitant EBV viral load.

## 3. Discussion

Propensity for lymphoma in ALPS is not well understood. The role of EBV, if any, is undefined. In the general population, EBV has been implicated as the causative agent in a number of lymphomas, including HL and certain DLBCL, albeit in only a proportion of cases [4]. Certain primary (e.g., Wiskott-Aldrich syndrome) [5] or secondary immunodeficiencies (e.g. transplant; HIV) are known to be at increased risk for EBV-LPD; again, only an unexplained select subgroup of patients is affected. In the original ALPS-associated lymphoma cohort at the National Institutes of Health, 10 of 130 subjects heterozygous for germline FAS mutation developed lymphoma: three (two of B cell origin, one of T cell) were EBV-positive on pathology by *in situ hybridization* (Table 1) [3]. Although the clinical penetrance of ALPS in subjects with germline FAS mutation is variable, lymphoma appeared to principally occur in only those who manifest some or all features of ALPS, an observation echoed by Neven et al. [6]. More recently, 20 subjects constituted the ALPS/lymphoma NIH cohort [2], of which 4 had EBV-associated disease [7], while Neven et al. [6] reported one EBV-positive case among their cohort of 7 subjects. Thus, ~15–30% of lymphomas in patients with ALPS appear to be EBV-related.

Surveillance for lymphoma in ALPS patients is challenging for several reasons: the waxing/waning course of lymphadenopathy; the difficulty in distinguishing nonmalignant lymphadenopathy from malignancy on metabolic imaging [9]; and limitations associated with repeat biopsies of multiple lymph nodes (e.g., feasibility; sampling error) during a potentially insidious course of illness that may span decades. While B-type symptoms (i.e., fever, night sweats, weight loss, and itching) may be suggestive, it may not always be present (as in this case) or may manifest only later in the lymphomagenesis process. Thus, there is a need for complementary tumor-specific biomarkers. Given that some lymphoma cases in ALPS patients appear to be EBV associated, monitoring EBV VL in blood may be a useful adjunctive tool to guide the need for more aggressive diagnostic investigations. Routine monitoring of EBV VL in blood is well established as a predictor and assessment of treatment response of PTLD, as well as other EBV-related malignancies [10]. In our case, initial lymph node biopsy demonstrated cells latently infected with EBV by EBER *in situ hybridization* but without evidence of lymphoma; there was no concomitant detectable EBV viremia. EBV VL was then monitored because of the historical diagnosis of “chronic EBV illness,” serendipitously illustrating a progressively increasing EBV VL in blood metachronous to lymphomatous transformation. Although no causality is implied, this case suggests that monitoring EBV VL in at least some ALPS patients may be a useful proxy test for certain types of lymphoproliferative malignancies. Nomura et al. recently reported the detection of EBV viremia in 3 patients initially diagnosed with CAEBV but subsequently found to be heterozygous for FAS mutations, suggesting that they, in fact, had ALPS [8]. However, in these patients, only a single EBV VL was reported and none had malignancy (although lymph node histopathology was not reported). Thus, if EBV VL proves clinically useful in diagnosing or predicting lymphoma in some patients with ALPS, it may be through dynamic increases on serial measurements, rather than from single values, as is seen in PTLD.

## 4. Conclusion

This case and the literature review suggest that patients with ALPS may have increased susceptibility to EBV-associated lymphomas. Further, this case fortuitously illustrates that at least in some patients with ALPS, serial monitoring of EBV VL may be a useful noninvasive biomarker to monitor for lymphomatous transformation. Further studies are needed to confirm these findings.

## Figures and Tables

**Table 1:** Clinical and laboratory profiles of ALPS patients with EBV lymphoma and/or EBV viremia.

Patient (reference; pedigree if available)	Country	Sex	Age of ALPS manifestations	Adeno-pathy	Spleno-megaly	Anemia	Thrombo-cytopenia	% DNTC∗	EBV^+^ Lymphoma	Lymphoma onset (years after ALPS onset)	EBV viremia	FAS mutation	Outcome
1^†^	Canada	M	4 years	+	+	+	−	15	Composite lymphoma (follicular lymphoma, diffuse large B-cell lymphoma, and classic Hodgkin's lymphoma)	30	+ (progressively increasing)	784A→T	Treated with chemotherapy. Alive.

2 [3] (26-II-4)	USA	M	2 years	+	+	+	+	0.7	Omental Burkitt's lymphoma	48	NR	973A→T	Treated with chemotherapy. Survived.

3 [3] (26-IV-5)	USA	M	10 months	+	+	+	NR	2.1	Classic Hodgkin's lymphoma (mixed cellularity)	7	NR	973A→T	Treated with chemotherapy; survived. Diagnosed with histiocytic sarcoma (at age 13); died from pulmonary hemorrhage after bone marrow transplant

4 [3] (45-III-2)	USA	M	5 years	+	+	+	+	11.7	Hodgkin's lymphoma	6	NR	779del11	Treated with chemotherapy. Outcome NR.

5 [3] (G3-III-4)	Germany	F	NR	+	+	−	+	75	T-cell lymphoma	2	NR	1009A→G	Treated with chemotherapy. Survived

6 [8]	Japan	M	3 months	+	+	−	+	10.4	NR	NR	+	IVS8 + 5G→T	Alive at 15 years old.

7 [8]	Japan	M	1	+	+	+	−	20.3	NR	NR	+	1020C→T	Alive at 14 years old.

8 [8]	Japan	M	2	+	+	−	+	12.5	NR	NR	+	1020C→T	NR

“+”: presence. “−”: absence. NR: not reported. ∗DNTC: double negative T cells = TCR *α*/*β* CD4- CD8-T cells.

^†^Current case; [3] Straus et al. Blood, 2001. [8] Nomura et al. Int J Hematol, 2011.

## List of Abbreviations

ALPS: Autoimmune Lymphoproliferative Syndrome
DNTCs: Double-negative T cells
EBV: Epstein-Barr virus
LPD: Lymphoproliferative disorder
HL: Hodgkin's lymphoma
VL: Viral load
PET-CT: Baseline positron emission tomography
EBER: EBV-encoded small RNA
DLBCL: Diffuse large B-cell lymphoma
R-CHOP: Rituximab, cyclophosphamide, doxorubicin,vincristine, and prednisone
PTLD: Posttransplant lymphoproliferative disorder
CAEBV: Chronic active Epstein-Barr virus.

## References

[1] Al-Herz W, Bousfiha A, Casanova JL (2011). Primary immunodeficiency diseases: an update on the classification from the international union of immunological societies expert committee for primary immunodeficiency. *Frontiers in Immunology*.

[2] Rao VK, Oliveira JB (2011). How I treat autoimmune lymphoproliferative syndrome. *Blood*.

[3] Straus SE, Jaffe ES, Puck JM (2001). The development of lymphomas in families with autoimmune lymphoproliferative syndrome with germline Fas mutations and defective lymphocyte apoptosis. *Blood*.

[4] Gandhi MK, Lambley E, Burrows J (2006). Plasma Epstein-Barr virus (EBV) DNA is a biomarker for EBV-positive Hodgkin’s lymphoma. *Clinical Cancer Research*.

[5] Amnueilaph R, Boongird P, Leechawengwongs M, Vejjajiva A (1973). Heroin neuropathy. *The Lancet*.

[6] Neven B, Magerus-Chatinet A, Florkin B (2011). Asurvey of 90 patients with autoimmune lymphoproliferative syndrome related to TNFRSF6 mutation. *Blood*.

[7] Venkataraman G, McClain KL, Pittaluga S, Rao VK, Jaffe ES (2010). Development of disseminated histiocytic sarcoma in a patient with autoimmune lymphoproliferative syndrome and associated rosai-dorfman disease. *American Journal of Surgical Pathology*.

[8] Nomura K, Kanegane H, Otsubo K (2011). Autoimmune lymphoproliferative syndrome mimicking chronic active Epstein-Barr virus infection. *International Journal of Hematology*.

[9] Rao VK, Carrasquillo JA, Dale JK (2006). Fluorodeoxyglucose positron emission tomography (FDG-PET) for monitoring lymphadenopathy in the autoimmune lymphoproliferative syndrome (ALPS). *American Journal of Hematology*.

[10] Kimura H, Ito Y, Suzuki R, Nishiyama Y (2008). Measuring Epstein-Barr virus (EBV) load: the significance and application for each EBV-associated disease. *Reviews in Medical Virology*.

